# Meeting Report: Estimating the Benefits of Reducing Hazardous Air Pollutants—Summary of 2009 Workshop and Future Considerations

**DOI:** 10.1289/ehp.1002468

**Published:** 2010-10-04

**Authors:** Maureen R. Gwinn, Jeneva Craig, Daniel A. Axelrad, Rich Cook, Chris Dockins, Neal Fann, Robert Fegley, David E. Guinnup, Gloria Helfand, Bryan Hubbell, Sarah L. Mazur, Ted Palma, Roy L. Smith, John Vandenberg, Babasaheb Sonawane

**Affiliations:** 1 National Center of Environmental Assessment, Office of Research and Development; 2 Office of Policy Analysis and Review, Office of Air and Radiation and; 3 National Center for Environmental Economics, Office of Policy, Economics and Innovation, U.S. Environmental Protection Agency, Washington, DC, USA; 4 Office of Transportation and Air Quality, Office of Air and Radiation, U.S. Environmental Protection Agency, Ann Arbor, Michigan, USA; 5 Office of Air Quality Planning and Standards, Office of Air and Radiation, U.S. Environmental Protection Agency, Research Triangle Park, North Carolina, USA; 6 Office of Science Policy, Office of Research and Development, U.S. Environmental Protection Agency, Washington, DC, USA; 7 National Center of Environmental Assessment, Office of Research and Development, U.S. Environmental Protection Agency, Research Triangle Park, North Carolina, USA

**Keywords:** air toxics, benefit analysis, economic valuation, exposure modeling, hazardous air pollutants

## Abstract

**Background:**

Quantifying the benefits of reducing hazardous air pollutants (HAPs, or air toxics) has been limited by gaps in toxicological data, uncertainties in extrapolating results from high-dose animal experiments to estimate human effects at lower doses, limited ambient and personal exposure monitoring data, and insufficient economic research to support valuation of the health impacts often associated with exposure to individual air toxics.

**Objectives:**

To address some of these issues, the U.S. Environmental Protection Agency held the Workshop on Estimating the Benefits of Reducing Hazardous Air Pollutants (HAPs) in Washington, DC, from 30 April to 1 May 2009.

**Discussion:**

Experts from multiple disciplines discussed how best to move forward on air toxics benefits assessment, with a focus on developing near-term capability to conduct quantitative benefits assessment. Proposed methodologies involved analysis of data-rich pollutants and application of this analysis to other pollutants, using dose–response modeling of animal data for estimating benefits to humans, determining dose-equivalence relationships for different chemicals with similar health effects, and analysis similar to that used for criteria pollutants. Limitations and uncertainties in economic valuation of benefits assessment for HAPS were discussed as well.

**Conclusions:**

These discussions highlighted the complexities in estimating the benefits of reducing air toxics, and participants agreed that alternative methods for benefits assessment of HAPs are needed. Recommendations included clearly defining the key priorities of the Clean Air Act air toxics program to identify the most effective approaches for HAPs benefits analysis, focusing on susceptible and vulnerable populations, and improving dose–response estimation for quantification of benefits.

Hazardous air pollutants (HAPs, or air toxics) are pollutants known to cause cancer or other serious health effects as well as environmental effects. The majority of air toxics originate from human-made sources, including mobile sources (e.g., automobiles) and stationary sources (e.g., factories). Under the Clean Air Act Amendments (CAAA) of 1990, the U.S. Environmental Protection Agency (EPA) regulates 187 HAPs from both stationary and mobile sources. HAPs are distinct from the criteria pollutants, such as ozone, carbon monoxide, particulate matter, sulfur oxides, nitrogen dioxide, and lead, that generally have much more extensive information about health effects and are regulated under other CAAA authorities. For major stationary sources, the act requires the EPA to set maximum achievable control technology (MACT) standards to reduce HAP emissions (CAAA 1990). After setting these standards, the U.S. EPA evaluates the residual risks from exposure to HAPs to determine if further regulation is warranted. For smaller stationary sources of HAPs (e.g., gasoline distribution, auto body refinishing), the EPA is required to set technology standards, which are known as generally available control technology (GACT). To control mobile source air toxics, the EPA is required to set technology and fuel standards for motor vehicles. Since 1990, the EPA has made significant progress in issuing standards under the CAAA to reduce emissions of air toxics both from stationary and mobile sources. To determine the impact of these efforts, Section 812 of the CAAA requires the EPA to perform periodic, comprehensive analyses of the costs and total benefits of programs implemented pursuant to the Clean Air Act (CAA 1970). The EPA has completed two of these analyses: a retrospective analysis in 1997 of the original CAA covering the period from 1970 to 1990 (U.S. EPA 1997) and a prospective analysis in 1999 of the incremental costs and benefits of the CAAA from 1990 to 2010 (U.S. EPA 1999). These and other studies have highlighted many of the challenges in estimating the benefits of reducing air toxics emissions.

## Challenges for Air Toxics Benefits Assessment

Benefits analysis for any environmental regulation requires estimating how individuals and regulated entities respond to the regulation, as well as estimating fate, transport, exposure, and effects of environmental chemicals. Thus, this type of assessment is subject to significant limitations and uncertainties. The U.S. EPA estimates benefits in regulatory impact analyses (RIAs) for rules that have a significant impact on the economy, typically based on a sequential analysis of emissions estimation modeling, air quality and exposure modeling, health and environmental effects estimation, and economic valuation. For major air toxics rules, the EPA has estimated benefits based on reductions in fine particulate matter (referred to as PM_2.5_) or ozone rather than those based directly on reductions of air toxics [Fann et al. 2009; Krupnick 2006; National Research Council (NRC) 2002)]. To estimate the benefits of reducing air toxics directly, there is a need for both near-term and long-term benefits assessment specific to the issues of air toxics reduction. Challenges related specifically to benefits analysis for air toxics include uncertainties in emissions information, air quality and exposure modeling, effects estimation, and economic valuation, as well as distribution considerations. These challenges are described below.

### Emissions

The U.S. EPA maintains an inventory of emissions for most of the 187 air toxics, although the data are less reliable than the inventory for the six criteria pollutants. The National Emissions Inventory is updated every 3 years and provides information from major stationary sources, smaller area stationary sources, on-road and nonroad mobile sources, and other sources (e.g., wildfires). Unlike criteria pollutants, reporting air toxics is not mandatory and varies by state. Therefore, some of these area sources have limited emissions information and are estimated through modeling. The U.S. EPA also publishes an annual Toxics Release Inventory (TRI) that requires facilities to report releases of 666 chemicals and chemical categories (including most of the air toxics) to air, land, and water (U.S. EPA 2010).

### Air quality and exposure modeling

The relationship between air emissions of toxics and human exposure is complex and often difficult to estimate. Air toxics vary widely in their sources, photochemical reactivity, and scale of spatial variability. In addition, exposure assumptions may differ significantly from actual individual exposures. For example, the typical assumption that people are exposed to the air outside their residences for 24 hr a day for a lifetime is used to simplify population analysis but introduces error in the exposure estimates, particularly when considered at the individual level (Isakov et al. 2009). Indoor sources may also be important components of individual exposure to air toxics and may be affected by emissions regulations (e.g., lawn and garden equipment in attached garages) (U.S. EPA 2007a; Weisel et al. 2002). Exposure assessment of average or cumulative exposures may miss significant acute exposures. The EPA National Air Toxics Assessment (NATA; U.S. EPA 2009b), which is currently available to the public with 2002 data, estimates ambient concentrations at the census-tract level (large scale) for mobile and area source emissions and at the census-block level (small scale) for major stationary source emissions]. The NATA then estimates inhalation exposure at the census-block level, which takes into account ambient exposures in various microenvironments, and calculates lifetime inhalation risks of cancer and noncancer health effects. The assessment does not provide estimates of ingestion risks that are important for certain air toxics such as mercury and lead (U.S. EPA 2001, 2009b).

### Health and environmental effects estimation

The list of HAPs to be regulated by the EPA is enumerated in the CAAA. For many chemicals on the list, the information on potential health effects is so limited that quantitative benefits analysis is not feasible (Goldstein and Carruth, 2003). This lack of information is in contrast to the criteria air pollutants for which there is extensive human exposure or epidemiological data on the health effects at ambient-exposure levels. For most of the air toxics, the available health information is based on animal studies. Therefore, reasoned assumptions about how these data relate to potential human health hazards are needed. The relatively smaller epidemiological data set for air toxics is based mostly on relatively high occupational exposures (e.g., for benzene) and has several limitations. These limitations include lack of information on multiple health end points across the life stages, susceptible populations, and differentially exposed groups. The potential for noncancer effects from exposure to air toxics is evaluated by comparing HAP concentrations with the reference concentration (RfC), which is a point estimate of the level expected to be without appreciable effects from chronic exposure in the population. The RfC definition does not incorporate any quantitative expression of risk. Furthermore, uncertainties related to quantitative values of risk for both cancer and noncancer health effects are rarely explored, understood, and characterized. For these and other reasons, characterizing the health effects of air toxics at ambient levels can be subject to a very high level of uncertainty; thus, using these health effects in economic benefits assessment is difficult. The recent NRC report, *Science and Decisions: Advancing Risk Assessment,* has provided recommendations applicable to issues related to benefits assessment of air toxics, including dose–response assessment, analysis of uncertainty and variability, default selection and use, and cumulative risk assessment (NRC 2009).

### Economic valuation

Standard economic analysis for pollution reduction starts with estimates of reduced mortality and morbidity effects (and other nonhealth effects as appropriate) (U.S. EPA 2000). Currently, benefits are estimated by aggregating reduced mortality risks to an expected number of deaths avoided, which are then multiplied by an aggregate willingness-to-pay (WTP) figure called the value of a statistical life (VSL). Reduced morbidity benefits are estimated similarly, when dose–response information is available to estimate the expected cases avoided, which are then multiplied by either an estimate of WTP to avoid the illness or an estimate of the cost associated with the treatment of that illness. Applying these methods to air toxics requires estimates of the mortality and morbidity effects. In the absence of these estimates, the benefits of toxics reduction may not be included in the analysis, resulting in a default assumption of zero benefits. In addition, people may be willing to pay for reducing toxics, even when the data on their effects are limited, to avoid perceived risks. The standard approach does not incorporate this willingness to pay.

### Efficiency versus distributional considerations

Regulations focused on air toxics seek not only to improve overall public health but also to address disproportionate risk in a segment of the population. The U.S. EPA must issue additional standards for sources after MACT if necessary to protect public health with an adequate margin of safety (CAAA 1990). In these cases, protecting the most exposed individuals rather than maximizing risk reduction over the entire exposed population may be the guiding objective. In fact, the CAAA specifically refer to reducing lifetime excess cancer risks to individuals most exposed to emissions from a source. Existing cost–benefit techniques are not intended to address tradeoffs between net benefits and distributional and equity considerations (Levy et al. 2009).

## Focus of 2009 Workshop

Discussions regarding best practices for estimating human health benefits from reductions in exposure to air toxics have been ongoing for years, but no consensus has been reached on methods that could be implemented for a broad selection of these toxics. Specifically, benefits analyses are inhibited by the lack of data relating exposures to health effects, uncertainties in extrapolating results from high-dose animal experiments to estimate human effects at lower doses, limited monitoring of ambient and personal exposure data, and insufficient economic research to support valuation of the health impacts often associated with exposure to individual air toxics.

Recognizing the uncertainties and research needs for many aspects of benefits assessment of reductions in air toxics, the U.S. EPA sponsored a workshop to explore the key issues related to health and environmental effects, economic valuation, and equity considerations (U.S. EPA 2009c). Highlights from these presentations are summarized below.

### Lessons learned from recent research

Risk assessors have been working on alternative ways to assess the benefits related to reducing air toxics. Workshop participants explored four case studies that used alternative approaches. These case studies focused on approaches that could be implemented with existing information, could potentially be conducted at a reasonable cost, were the least likely to introduce bias, and were the most scientifically defensible. They included developing methodologies for data-rich HAPs (e.g., benzene) with a broader application to other HAPs, using animal data to estimate a dose–response curve for adverse health effects in humans (e.g., acrolein), using dose-equivalent relationships between chemicals with similar adverse health effects (e.g., toluene), and analyzing HAPs similar to that performed for criteria pollutants (e.g., lead) (Table 1). In addition to these four case studies, the workshop participants considered environmental quality in relation to changing community composition and equity tradeoffs. These alternative methodologies and key issues suggest potential options for analyzing benefits from air toxics reductions and highlight continued challenges in the field. The case studies are described in more detail below and summarized in Table 1.

#### Benefits of benzene reductions in Houston, Texas

In a case study designed to estimate the benefits of reducing benzene for a three-county area around Houston, Texas, scientists quantified changes in individual risk using air quality and exposure models and translated these changes into monetized benefits [Industrial Economics, Inc. (IEc) 2009]. Benzene was used as an example of a data-rich air toxic pollutant to develop a methodology that could then be applied more widely. The case study modeled benzene exposures and health impacts using two scenarios: One assumed that all regulatory programs affecting benzene emissions were enacted in response to CAAA, and one assumed that no additional controls were implemented beyond the requirements existing in 1990. The difference in emissions between these two scenarios provided the basis for estimating the health benefits related to the reduction of benzene concentrations in Houston, Texas, because of the CAAA. As part of the Houston study, scientists examined the incidence of leukemias to estimate the health benefits from reductions in benzene exposure. The results demonstrated a decrease in fatal and nonfatal leukemias between 1990 and 2020. To measure the health outcomes, researchers used the VSL to monetize the avoided fatal leukemias and the WTP to monitize the nonfatal leukemias. This case study represents the uncommon situation where relatively complete information is available (e.g., human health evidence, air quality monitoring data) to estimate the health effects of air toxics and the benefits of reducing them. However, uncertainties and limitations of the approach used in this case study include large variations in the results because of the sensitivity of the model to unputs; for example, varying dose–response estimates or alternative cessation lags; the possibility that other health end points related to benzene exposure were not examined; and the limited age groups studied in the cohort. The approach used in the Texas case study was data- and resource-intensive and may be difficult to expand to other air toxics.

#### Estimating risk from acrolein

The analysis of acrolein risks for respiratory effects examined the applicability of using dose–response modeling of animal data to estimate the benefits to humans. Using data from a study in rats exposed to acrolein (Costa et al. 1986), Woodruff et al. (2007) evaluated two end points that are indicators of reducded lung function. These end points were selected based on the presence of a significant dose-related trend, on data that was amenable to modeling, and on the relevance to changes in lung function in humans. Acrolein concentrations from the rat study were converted into human equivalent concentrations using U.S. EPA standard methods (U.S. EPA 2003), and the data for each end point were modeled using EPA Benchmark Dose Software (U.S. EPA 2009a). Increased risk was then estimated as the proportion of the population that would experience a change in adverse lung function, considering ambient concentrations in the United States estimated in NATA. This case study represents the more typical situation where relatively little information is available (e.g., animal toxicology data) to estimate air toxics health effects and the benefits of reducing them.

#### Cost of neurobehavioral effects of toluene exposure

Benignus et al. (2005) estimated the economic benefits of reducing exposure to toluene by comparing these neurobehavioral effects with those of well-studied ethanol ingestion. The investigators used dose-equivalent relationships to estimate the costs of intoxication. Because many air toxics have effects on the nervous system that are very similar to those of ethanol, Benignus et al. (2005) quantified behavioral effects of toluene and ethanol in human subjects using a meta-analysis of studies from the peer-reviewed literature that measured the effects of the chemicals on choice reaction time (CRT). CRT is a two-choice reaction time test that is useful for assessing general alertness and motor speed. Models were used to estimate internal doses of ethanol and toluene associated with effects on CRT from exposure parameters provided in the published literature. This case study represents the scenario where relatively complete information is available for one chemical (ethanol) to estimate air toxics health effects and assess the benefits of reducing the effects for other chemicals with less information, such as toluene. The uncertainties of this method include the quality of the exposure data and the extent of the mechanistic similarity between the observed effects of the two compounds.

#### Human health benefits assessment for lead standards

The final case study was an analysis of the human health and welfare benefits associated with attaining alternative levels for the lead National Ambient Air Quality Standards (NAAQS). This case study was part of the RIA to estimate the expected benefits and costs of attaining a new NAAQS (U.S. EPA 2008). This was an example of methodologies that can be applied for environmental chemicals with extensive epidemiological data and highlighted the complications introduced by some key data gaps that may exist even for data-rich chemicals. The U.S. EPA quantified the monetary value of lead-associated changes in intelligence quotient (IQ). This analysis followed four basic steps. First, the U.S. EPA estimated the change in ambient lead resulting from attainment of alternate NAAQS levels, relative to baseline ambient lead levels in 2016. Then the EPA applied air-to-blood ratios to quantify the change in blood lead as a function of exposure to ambient lead. The third step was to apply relevant epidemiological studies to quantify the population-level change in IQ points. Finally, the EPA monetized the change in IQ points by using economic valuation functions to measure the foregone lifetime earnings per lost IQ point. As part of a sensitivity analysis to test the importance of key model inputs, the analysis concluded that total benefits were highly sensitive to the air quality estimates, the discount rate, and the air-to-blood ratio. The U.S. EPA approach to estimating ambient lead-related benefits may serve as a useful model for future analyses of air toxics with noninhalation exposure pathways.

#### Additional presentations related to air toxics benefits analysis

Two additional presentations addressed distributional considerations for benefits analysis. The first, an analysis of changing environmental quality on community composition, provided a simple model analysis of the relationship between TRI emissions and low-income communities and showed that reducing exposure in these communities would not necessarily have favorable distributional effects (Walsh and Banzhaf 2009). Using the example of the impact of TRI emissions reductions in California, it was concluded that estimating the true effects of reductions of air toxics on local communities requires a more thorough understanding of the relationships among environmental improvements, real estate markets, and demographics. This study underscored the role of sociological factors in air toxics benefits analyses. In the second study, Levy et al. (2007) highlighted recent research that evaluated the efficiency–equality tradeoffs in health benefits analysis; they used a modeling framework that may have potential implications for air toxics emissions reduction. These tradeoffs are between the magnitude of (efficiency) and the distribution of the health benefits (equality). The challenge in their analysis was to find a simple, meaningful indicator that could capture the distribution of the benefits of pollution control from a source or set of sources. This analytical framework could be applied to capture the distribution of baseline risk from air toxics and the distribution of risk postcontrol. For this framework to be useful, multiple well-defined and realistic control scenarios would have to be developed. This study demonstrated that approaches for incorporating equity considerations may have applications for air toxics benefits assessment.

Three particular issues relating specifically to the estimation of air toxics benefits reduction were also explored in more depth: valuing reductions in individual risks, WTP for reducing air toxics, and alternatives to pollutant-by-pollutant dose–response estimation. Valuing large reductions in individual mortality risks focused on evaluating whether the magnitude of estimated risks being reduced requires new valuation methods or adjustments in current values to reflect the extent of risk changes. Labor market estimates of VSL provide the wage–risk tradeoffs for small risks. The degree to which these values are applicable to a particular benefits analysis depends, in part, on the magnitude of the risk reduction (Viscusi 2009).

WTP analysis for the large number of air toxics creates a challenge when using a damage-function approach to assess benefits, as it requires a dose–response relationship for each HAP. Key issues for valuation of reduced air toxics risks include what benefits to value, whether altruistic values should be included, whether to value subjective or objective risks, and whether valuation results could be applied to different policies (Cropper and Krupnick 2009). Ideally, a WTP approach would separate risk assessment from valuation.

A major issue for assessing HAPs is that the measures for estimating noncancer health risks (e.g., RfC) do not lend themselves to quantification of risk reductions for benefit analysis (Hattis et al. 2002). Methods to overcome the barriers to the quantification of noncancer health risks were discussed. More specifically, research that focuses on estimating risks without extensive chemical-specific toxicity information, creating a framework for understanding and quantifying the uncertainties created by missing data that can be the basis for value-of-information analysis, and facilitating comparisons and priority setting for controlling exposure to different air toxics is needed (Hattis and Lynch 2009).

### Road map on benefits assessment for air toxics

Air toxics benefits assessment has focused on a limited number of pollutants based on available dose–response and exposure information, generally on a worst-first basis. One main purpose of the workshop was to discuss other near-term benefits assessment options as laid out in the draft roadmap (U.S. EPA 2009c). Although the roadmap also listed many areas for long-term research, the workshop participants focused more on the five near-term options in the roadmap:

- *Qualitatively assess air toxics reduction benefits with no attempt at quantification.* This approach gives risk assessors the flexibility to make a qualitative case for air toxics control, while not exposing the analysis (or the regulation it supports) to the drawbacks and uncertainties associated with estimating benefits in quantitative terms from air toxics reductions based on the current state of the science. A concern with this approach is that some will assume that benefits are negligible rather than unquantifiable, and therefore the value of the air toxics program and its regulations may be underestimated.
- *Use the existing frameworks to sketch out minimum benefits from a national perspective.* This approach would build on work that is ongoing regarding benefits analysis looking pollutant by pollutant. For example, risk assessors could use the benzene methodology to estimate benefits of reducing benzene exposure on a wider geographic scale and to estimate benefits of reducing other carcinogens with human data on inhalation exposures (e.g., 1,3-butadiene); to adapt the acrolein methodology to estimate effects of other priority inhalation noncancer pollutants (e.g., manganese); and to use the lead methodology to estimate benefits from other noninhalation risks (e.g., mercury). This approach would build on peer-reviewed work and provide an assessment of fatal and nonfatal cancer effects for certain pollutants, using standard benefits methods. It could also focus on the pollutants that risk assessors believe are driving the majority of the risks from a national perspective. However, this approach would not address some of the challenges laid out earlier, such as possible underestimation of health effects from hot spots or short-term exposures and effects of interactions among pollutants on adverse health outcomes in a population across life stages and/or in disproportionately exposed populations.
- *Use NATA or other existing modeling tools to pursue national or regional or local analysis focusing on reduction of individual risk levels.* This approach has been used with the 1999 NATA framework as part of the 2007 Mobile Source Air Toxics Rule (U.S. EPA 2007b). In that rule, air toxics modeling was done for 1999 and several future years, with and without controls. The 1999 NATA was modified to account for the pollutant gradients near roads, analyzing 19 mobile source air toxics. The analysis included estimates of monetized benefits from PM reductions that also occurred as a result of the rule but did not monetize reductions in toxics risk. This is primarily because the NATA framework is not adequate for extrapolation to incidence estimations or benefits assessment (U.S. EPA 2001, 2007b). The model has several limitations, including the inability to estimate specifics for different age groups and the lack of accounting for indoor sources and potentially important exposure scenarios. The strengths of this approach include the ability to examine multiple pollutants at the same time and look across various geographic areas to estimate impacts on individual risk levels.
- *Estimate benefits of air toxics emissions reductions in conjunction with the criteria air pollution program*. Many regulations and implementation actions to meet criteria air pollution goals affect the same sources that emit significant air toxics. In some cases, the emissions that contribute to ambient concentrations of ozone, PM, and other criteria air pollutants are also air toxics. To date, with the significant exception of mobile source regulations of volatile organic compound and PM precursor emissions, the U.S. EPA has not assessed the air toxics impacts of the criteria air pollutant programs. Because these criteria pollutant programs are often very broad in nature, covering many sources and geographic areas, the cumulative impact on air toxics may be large. At the very least, risk assessors could estimate changes in population-weighted concentrations of air toxics, even in the absence of appropriate concentration–response functions linking concentrations with health end points. This approach would provide a consistent air-quality framework for integration with other benefits analyses. However, without concentration–response functions, this approach still does not provide quantified health impacts or economic benefits.
- *Expand benefits assessment efforts to include equity considerations*. As noted earlier, there is an emerging literature on addressing equity considerations in addition to traditional analyses of efficiency. These approaches use statistical measures of inequality to determine the change in the population distribution of air toxic risks. These changes in equity can then be compared with changes in total public health benefits to identify possible complementarities in health benefits and equity or the tradeoffs between health benefits and equity.

## Conclusions

The workshop provided new perspectives on benefits assessment of air toxics from participants with a wide range of expertise. Some key recommendations that emerged on moving forward on benefits assessment include the following:

- A clear definition of the purpose(s) of HAPs benefits analysis to frame long-term research priorities is needed. The near-term and long-term approaches would vary based on whether the focus should be on reducing air toxics for the general population, for hotspots and regions of interest, or for the most susceptible populations. A value-of-information approach was recommended to prioritize future research based on what would have the greatest impact on benefit assessment outputs and what would reduce uncertainties most effectively.
- Grouping by emissions sources would address more closely the issue of hotspots or highly exposed populations, whereas grouping by chemical class would allow the use of toxicology and health data for well-studied chemicals to inform those with more limited information but of similar structure or mode of action.
- Different population groups may be of particular concern for air toxics exposures, depending on the spatial and temporal distribution of exposures. Therefore, accounting for the heterogeneity in temporal and spatial distribution, specifically for emissions and receptors (e.g., children and other vulnerable populations) is critical for benefits analysis.
- The National Health and Nutrition Examination Survey work on exposure distributions could be used to inform both exposures and health end points (CDC 2010).
- Analytical methods to define and measure equity considerations should be better supported.
- There is a critical need to improve dose–response estimations, potentially through use of models for probabilistic estimation of noncancer health risks.
- More research on surveillance and biomonitoring is needed to understand more fully how reductions in air toxics related to specific health effects.
- More support for research on the use of predictive biomarkers of exposure and health effects would allow for an early measurement of the impact in reduction of air toxics.

The above key recommendations provide some specific steps forward in advancing analysis of the benefits from air toxics reductions and suggest some future studies to inform many of the challenges in this field.

## Figures and Tables

**Table 1:** Overview of case studies that explored various methodologies for benefits analysis of regulating air toxics.

Chemical name	Dose–response data	Health end point(s) analyzed	Methods for risk evaluation	Methods for valuation	Size of benefit/potential utility	Uncertainties	Reference
Benzene	Human epidemiological studies (occupational cohort)	Leukemias (fatal and nonfatal)	Life table approach	VSL for fatal leukemias; approximation of willingness to pay for nonfatal leukemias	Model demonstrated percent reduction in risk by 2020 from 72 to 98% depending on county	Model sensitive to inputs; only quantified leukemias and not other health effects; low-dose extrapolation issues	IEc 2009
Acrolein	Analysis of existing animal studies with dose conversion to human equivalents	Respiratory effects (noncancer)	Dose–response functions combined with modeled ambient acrolein concentrations to estimate risks of adverse effects	Not carried out in this study; further analysis needed to relate respiratory outcomes observed in animals to likely human effects	Method used animal data to inform benefits of reduction in exposure in human populations	Assumptions based on low-dose extrapolation and human interpretation of end point (adverse effects as general indicators of potential human lung function changes)	Woodruff et al. 2007
Toluene	Meta-analysis of human epidemiology studies on toluene and ethanol effects	Neurobehavioral effects	Dose-scaling comparison for acute effects of toluene exposure with those of ethanol intake	Effect of toluene scaled to that of ethanol; used costs of ethanol intoxication (e.g., automobile crashes)	Comparison with another toxicant with the same mechanism of action that has been monetized can yield a quantitative benefits estimate	Assumptions needed for mechanisms of both toxicants and dose–response comparisons	Benignus et al. 2005
Lead	Human epidemiology studies	IQ	Model changes in lead air quality for the exposed population and translated these into changes in blood lead	Present value of lifetime loss in earnings per IQ point lost	Provide a credible estimate of the human health benefits of attaining alternate lead NAAQS	Uncertainties in air-lead to blood-lead relationship; valuation method had substantial limitations	U.S. EPA 2008

Abbreviations: IQ, intelligence quotient; NAAQS, National Ambient Air Quality Standards.
